# Thymic Carcinoma Presenting as Diffuse Bone Marrow Infiltration Mimicking Lymphoma

**DOI:** 10.7759/cureus.114862

**Published:** 2026-08-20

**Authors:** Krystel P Mateus Farias, Edgar R Reyes Mayaute, Walmer M Tarazona Cervantes, Carmen R Pozo Morales, Ronald J Alvarez Guzman, Roxana V Murrugarra Meza, Carlos Valladares

**Affiliations:** 1 Institute of Research in Biomedical Sciences, Universidad Ricardo Palma, Lima, PER; 2 Department of Internal Medicine, Hospital Nacional PNP Luis N. Sáenz, Lima, PER; 3 Department of Pulmonary and Critical Care, University of Miami Leonard M. Miller School of Medicine, Jackson Memorial Hospital, Miami, USA

**Keywords:** bone marrow infiltration, bone marrow metastasis, diagnostic challenge, differential diagnosis, immunohistochemistry, lymphoma mimic, mediastinal mass, mediastinal neoplasms, thymic carcinoma, thymic epithelial tumors

## Abstract

Thymic epithelial tumors (TETs) represent the most common primary neoplasms of the anterior mediastinum, yet thymic carcinoma (TC) stands out as an exceptionally rare and biologically aggressive subtype. Unlike thymomas, TCs exhibit marked cytologic atypia, early invasive potential, and a high predisposition for extrathoracic metastasis. We report the case of a 59-year-old male who presented with a seven-month history of progressive, refractory lumbar pain and functional limitation. Initial clinical and laboratory evaluation revealed normocytic anemia, leukocytosis, elevated inflammatory markers, and increased lactate dehydrogenase and beta-2 microglobulin levels. These findings, combined with systemic bone marrow infiltration observed on spinal magnetic resonance imaging, initially suggested a lymphoproliferative disorder. A subsequent thoracic computed tomography scan identified an anterior mediastinal mass with lymphadenopathy. A bone marrow biopsy evidenced infiltration by a poorly differentiated malignant neoplasm, and an immunohistochemical profile of the mediastinal mass confirmed the diagnosis of stage IVb TC, showing positivity for pan-cytokeratin, tumor protein p63, cluster of differentiation 5 (CD5), epithelial membrane antigen (EMA), and cluster of differentiation 117 (CD117), with a Ki-67 proliferation index of 20%, while remaining negative for hematolymphoid, neuroendocrine, and lung-specific markers. The patient was managed through a multidisciplinary approach with systemic palliative chemotherapy. This case highlights a critical diagnostic challenge where TC mimicked a hematological malignancy. The systemic dissemination of TC, particularly the diffuse bone marrow infiltration, is a rare clinical phenotype that obscures the primary mediastinal origin. Our report emphasizes the necessity of including TC in the differential diagnosis of patients presenting with refractory bone pain and systemic hematological abnormalities, even in the absence of respiratory symptoms. Early diagnostic recognition through strategic immunohistochemical profiling is imperative to avoid clinical bias, prevent the initiation of inappropriate therapies, and facilitate a prompt, evidence-based management strategy for patients with advanced, non-resectable disease.

## Introduction

Thymic epithelial tumors (TETs) represent the most frequent primary lesions of the anterior mediastinum; however, thymic carcinoma (TC) constitutes an exceptionally rare and biologically aggressive subtype, accounting for barely 5%-10% of all TETs [1]. Unlike thymomas, TC is characterized by marked cytologic atypia, early invasive behavior, and a high predisposition to extrathoracic metastasis, with a global incidence estimated at 0.3-0.6 cases per million inhabitants per year. Crucially, diffuse bone marrow infiltration is an exceptionally uncommon presentation of TC that can closely resemble hematological malignancies, representing the most distinctive feature of this case [2,3].

Epidemiologically, this entity predominates in the fifth and sixth decades of life, with a slightly higher prevalence in males, as observed in the present case [4]. TC presents a significantly higher rate of metastasis at the time of diagnosis compared to thymomas, reaching up to 40-50% of patients in advanced stages [5]. The most frequent sites of extrathoracic dissemination include the lungs (30%), liver (25%), and the skeletal system (20%), followed in smaller proportions by extrathoracic lymph nodes and the central nervous system. An initial presentation dominated by diffuse bone marrow involvement is exceptionally rare and poses a major diagnostic challenge due to its mimicry of primary hematological disorders [6,7].

Clinical presentation is usually insidious; approximately 50% of patients are asymptomatic in the early phases, while the remainder present with symptoms derived from the compression of mediastinal structures, such as cough, dyspnea, or superior vena cava syndrome [8,9]. Nevertheless, metastatic bone presentation as an initial symptom is infrequent and represents a major diagnostic challenge, as it may mimic hematological malignancies or metastases from prostatic or pulmonary adenocarcinoma [10].

The relevance of this case lies in the complexity of the differential diagnosis. The elevation of biomarkers such as beta-2 microglobulin and lactate dehydrogenase (LDH), together with mediastinal lymphadenopathy, frequently orients the initial suspicion toward primary mediastinal lymphomas [11]. Given its extreme rarity and the absence of standardized management guidelines for advanced stages (Masaoka IVb), reporting atypical presentations is fundamental. This case highlights the diagnostic complexity of TC mimicking a hematological malignancy and emphasizes the indispensable role of immunohistochemistry, specifically markers such as CD5 and CD117 (c-KIT), to confirm the diagnosis, differentiate it from other squamous cell carcinomas and lymphomas, and guide appropriate clinical management [12,13].

## Case presentation

A 59-year-old mestizo male with a history of benign prostatic hyperplasia (treated with tamsulosin 0.4 mg/day for 10 years) and post-traumatic stress disorder, anxiety, and depression (managed with sertraline) presented for evaluation. He denied relevant family history or drug allergies.

​The patient reported a seven-month history of insidious, progressive lumbar pain, non-mechanical in nature, radiating to the gluteal region and the anterior aspect of both thighs. This pain was associated with progressive functional limitation and did not respond to self-medication with tramadol. Notably, the patient denied constitutional symptoms, such as fever, unexplained weight loss, or night sweats. Furthermore, despite the later findings of a mediastinal mass, he exhibited no respiratory or compressive symptoms, specifically denying cough, dyspnea, chest pain, dysphagia, or signs of superior vena cava syndrome. Upon admission, the patient was hemodynamically stable and in fair general condition, with a Glasgow Coma Scale score of 15/15. Physical examination revealed tenderness on palpation of the lower lumbar region (L4-L5), without focal neurological deficits; the straight leg raise test was negative. Ecchymosis was observed in the posterior cervical region, attributed to previous alternative therapies.

Laboratory studies evidenced mild normocytic anemia, persistent leukocytosis, elevated C-reactive protein, elevated fibrinogen, and a slightly increased erythrocyte sedimentation rate. Furthermore, elevated LDH, hyperferritinemia, and increased beta-2 microglobulin were noted, with prostate-specific antigen levels remaining within the normal range (Table 1).

**Table 1:** Laboratory findings on admission ​CRP: C-reactive protein; LDH: lactate dehydrogenase; ESR: erythrocyte sedimentation rate; g/dL: grams per deciliter; cells/µL: cells per microliter; mg/L: milligrams per liter; ng/mL: nanograms per milliliter; U/L: units per liter; mm/h: millimeters per hour; mg/dL: milligrams per deciliter ​Laboratory investigations demonstrate marked leukocytosis, normocytic anemia, and significantly elevated inflammatory and acute-phase reactants.

Test	Result	Reference range
Hemoglobin	10.5 g/dL	12-16 g/dL
White blood cell count	15,910 cells/µL	4,500-10,500 cells/µL
C-reactive protein (CRP)	96 mg/L	<5 mg/L
Ferritin	1100 ng/mL	30-300 ng/mL
β2-microglobulin	2.81 mg/L	0.8-2.2 mg/L
Lactate dehydrogenase (LDH)	417 U/L	140-280 U/L
Erythrocyte sedimentation rate (ESR)	25 mm/h	0-20 mm/h
Fibrinogen	642 mg/dL	200-400 mg/dL

**Figure 1:**
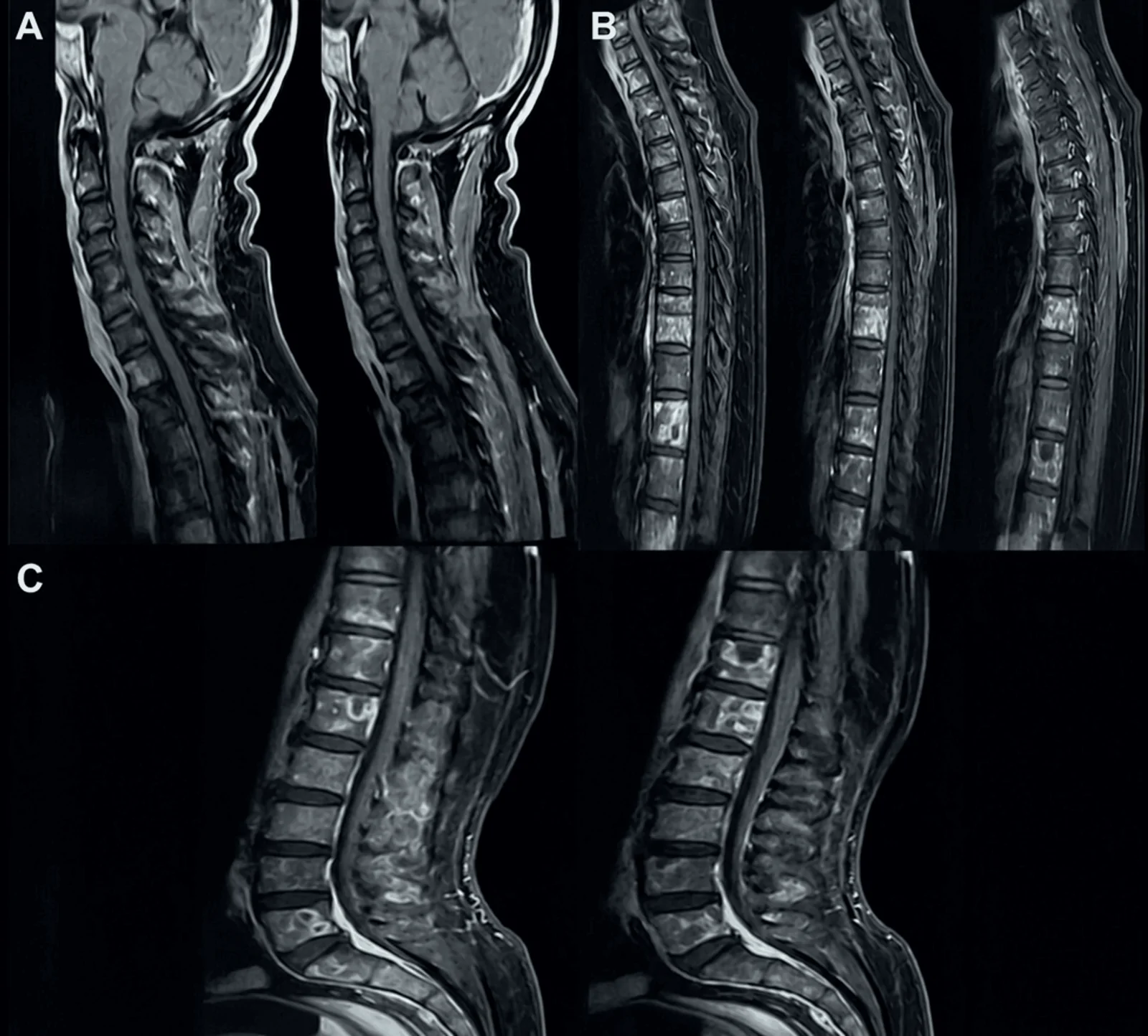
Sagittal STIR MRI of the cervical (A), thoracic (B), and lumbosacral (C) spine demonstrating diffuse hyperintense marrow lesions involving multiple vertebral bodies, consistent with extensive osseous metastatic disease and diffuse bone marrow infiltration

Bone scintigraphy showed multiple hypermetabolic foci in the calvarium, ribs, and right sacroiliac joint, suggestive of metastatic disease. Magnetic resonance imaging (MRI) of the cervical, thoracic, and lumbosacral spine confirmed diffuse bone marrow infiltration with multiple lytic lesions (Figure 1).

**Figure 2:**
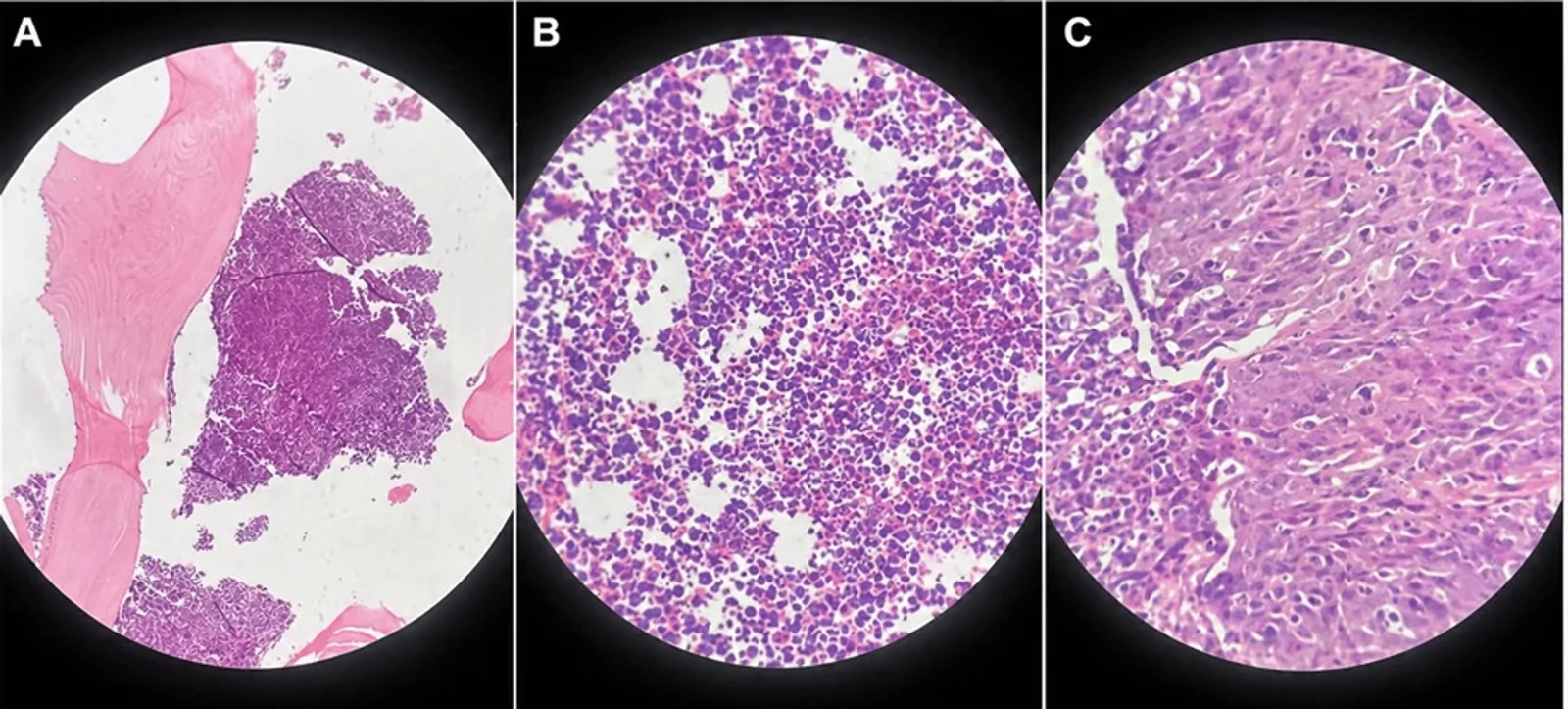
Bone marrow biopsy (hematoxylin and eosin stain) (A) Panoramic view evidencing medullary architecture. (B) Areas of hematopoietic tissue with preserved characteristics. (C) Foci of infiltration by a malignant dysplastic neoplasm characterized by marked cellular atypia and structural disorganization.

​Non-contrast chest computed tomography (CT) revealed an anterior mediastinal lymphadenopathic conglomerate measuring 46 x 27 x 51 mm, alongside multiple mediastinal lymphadenopathies, including pretracheal nodes (measuring up to 15 x 9 mm and 14 x 10 mm) and aortopulmonary window nodes (measuring up to 17 x 10 mm and 15 x 10 mm). Abdominal CT showed hepatomegaly, simple renal cysts (Bosniak I), grade II prostatic enlargement, and degenerative bone changes, without significant retroperitoneal lymphadenopathy. Given the initial suspicion of a lymphoproliferative syndrome, a bone marrow biopsy was performed, revealing infiltration by a poorly differentiated malignant neoplasm (Figure 2).

**Figure 3:**
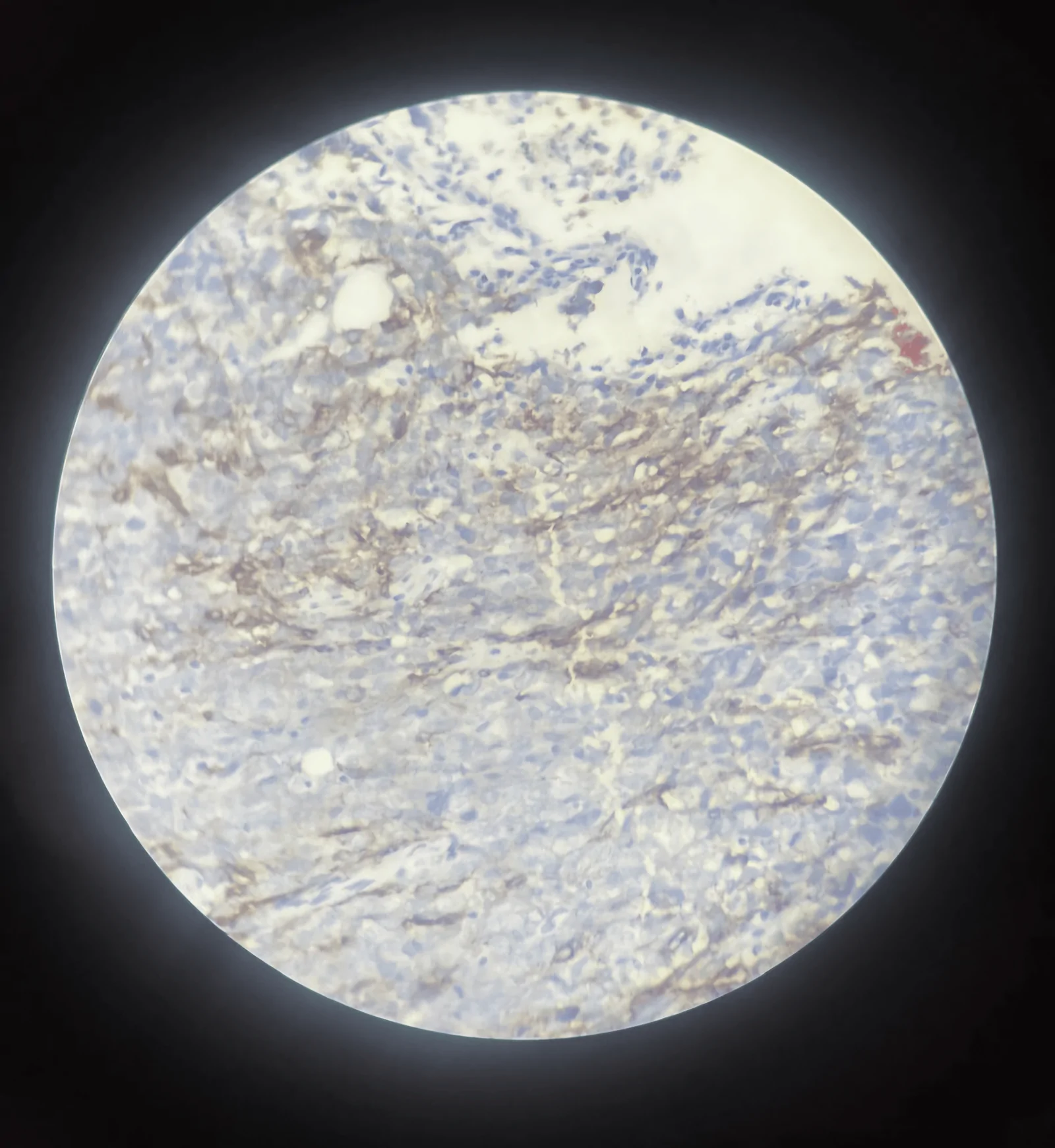
Pan-cytokeratin immunohistochemical stain demonstrating diffuse positivity in the tumor cells of the anterior mediastinal mass, confirming epithelial differentiation

**Figure 4:**
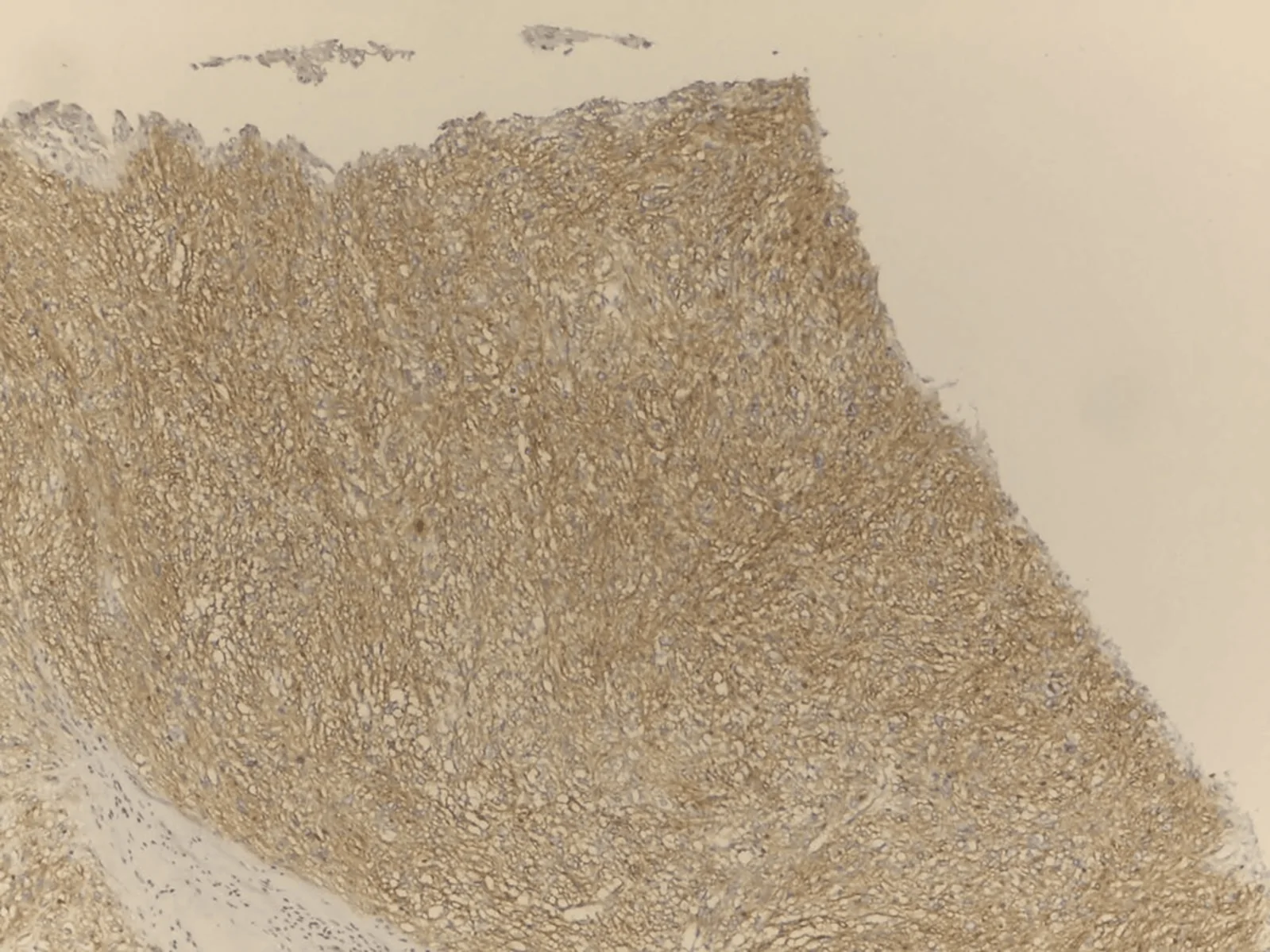
Immunohistochemistry for CD117 (c-KIT) in the mediastinal lymph node biopsy High-resolution view (40x) demonstrating diffuse sheets of neoplastic epithelial cells infiltrating the lymph node parenchyma, with strong and diffuse immunoreactivity in a distinct membranous and cytoplasmic pattern.

Due to the initial suspicion of a primary hematological malignancy given the diffuse bone marrow infiltration and elevated LDH/beta-2 microglobulin, a bone marrow biopsy was performed first in March 2026. Following atypical/inconclusive results, a core needle biopsy of the anterior mediastinal mass was performed one month later, in April 2026, showing high-grade epithelial tumor proliferation with desmoplastic stroma. Immunohistochemical study revealed positivity for pan-cytokeratin, tumor protein p63, cluster of differentiation 5 (CD5), cluster of differentiation 117 (CD117), and epithelial membrane antigen (EMA) (Ki-67 20%) (Figures 3-4, Table 2).

**Table 2:** Immunohistochemical profile of the mediastinal mass The immunoprofile confirms an epithelial lineage (pan-cytokeratin+, EMA+) and is highly suggestive of thymic carcinoma (CD5+, CD117+). The negativity for lymphoid (CD45, CD3, CD20), neuroendocrine, and lung markers excludes other diagnostic possibilities.

Marker	Result	Interpretation/Significance
Pan-cytokeratin	(+)	Confirms epithelial lineage
p63	(+)	Marker for basal/thymic epithelial cells
CD5	(+)	Highly suggestive of thymic carcinoma (rare in other sites)
EMA	(+)	Confirms epithelial origin
CD117 (c-kit)	(+)	Frequently positive in thymic carcinomas
Ki-67	20%	Indicates a moderate cell proliferation rate
Chromogranin	(-)	Rules out neuroendocrine differentiation
Synaptophysin	(-)	Rules out neuroendocrine differentiation
CD45, CD3, CD20	(-)	Rules out lymphoma (lymphoid lineage)
CD56	(-)	Helps rule out neuroendocrine/NK tumors
S100	(-)	Rules out tumors of neural origin or melanoma
TTF-1	(-)	Helps exclude primary lung adenocarcinoma

**Figure 5:**
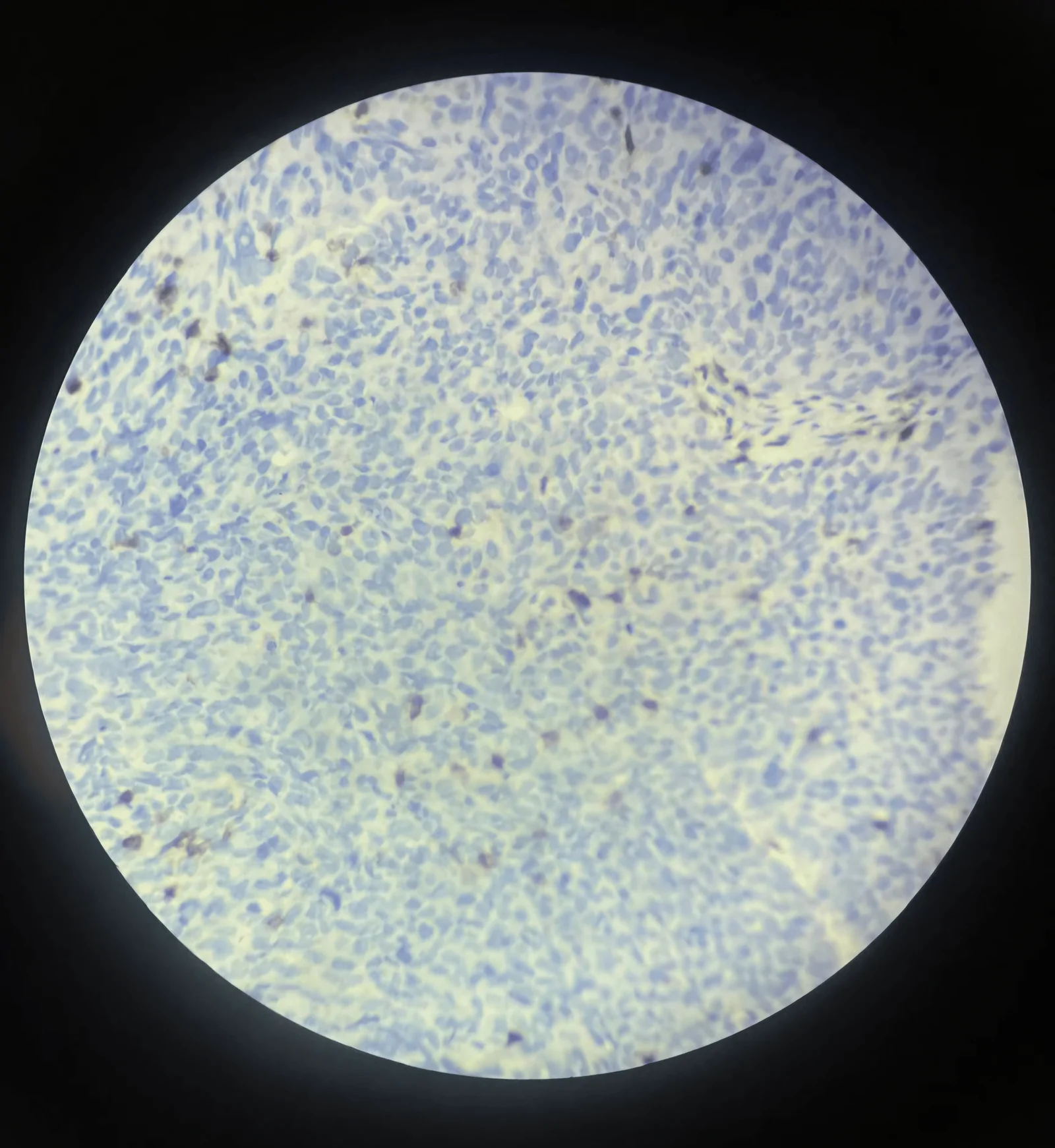
Negative CD45 immunostaining in the neoplastic cells of the anterior mediastinal mass, with no evidence of tumor lymphoid infiltration, excluding a hematolymphoid origin and supporting the diagnosis of thymic carcinoma

Samples were negative for hematolymphoid markers (CD45, CD3, CD20), neuroendocrine markers (chromogranin, synaptophysin, CD56), and markers of other origins (TTF-1, S100). These findings, combined with the morphology and mediastinal location, were consistent with stage IVb (Masaoka-Koga) metastatic TC (Figure 5).

The patient's management was multidisciplinary. A curative surgical approach was ruled out due to the extent of metastasis. Medical oncology initiated first-line palliative chemotherapy with a carboplatin/paclitaxel regimen, supplemented by high-protein nutritional support. For severe lumbar pain, a scheduled analgesic therapy with tramadol and orphenadrine was established, supplemented with intravenous tramadol and dimenhydrinate for breakthrough pain. Additionally, gastrointestinal support with simethicone and levosulpiride was prescribed, and the patient continued his usual medication (sertraline and tamsulosin) with the addition of nocturnal atorvastatin. Currently, the patient remains under close outpatient follow-up by oncology and palliative care.

## Discussion

We present the case of a 59-year-old male with progressive and refractory lumbar pain of seven months' duration, whose initial workup revealed diffuse bone and marrow infiltration along with an anterior mediastinal mass with associated lymphadenopathy. Histopathological and immunohistochemical evaluation confirmed the diagnosis of metastatic TC (Masaoka-Koga stage IVb), illustrating an exceptionally atypical presentation that mimicked a lymphoproliferative syndrome and delayed the recognition of this highly aggressive neoplasm.

Unlike what is described in the literature, where TC manifests predominantly with intrathoracic symptoms, such as dyspnea, cough, or chest pain secondary to the mediastinal mass effect [14,15], our case debuted exclusively with chronic refractory lumbar pain. Although atypical presentations or incidental findings in thymic neoplasms have been reported, these usually correspond to localized tumors discovered during radiological studies for other causes, and not to initial manifestations of systemic and symptomatic metastatic disease [16]. Similarly, although bone dissemination is documented in advanced stages, it usually occurs in the context of a known primary tumor or accompanied by thoracic symptoms [17]. The clinical value of this report lies in the diffuse bone marrow infiltration as an initial presentation, an exceptionally infrequent form of presentation that masks the solid neoplasm under a musculoskeletal or hematological guise. This finding not only expands the known clinical spectrum of TC but also underscores the critical need to include it in the differential diagnosis of refractory bone pain associated with abnormal analytical warning signs, even in the absolute absence of initial respiratory symptoms.

​From a diagnostic standpoint, diffuse bone lesions with elevated LDH, hyperferritinemia, and beta-2 microglobulin initially suggested a hematological malignancy or metastatic adenocarcinoma. Normal PSA levels and the absence of primary pulmonary lesions ruled out prostatic and lung carcinomas. Evaluation of the anterior mediastinal mass narrowed the differential from germ cell tumors and indolent thymomas to TC, given its invasive nature and extensive osseous dissemination.

The relationship between the bone marrow and mediastinal tissue evaluations was critical in establishing a unified diagnosis. The initial bone marrow biopsy demonstrated malignant infiltration by epithelial carcinoma rather than a primary hematological disorder. However, given its non-conclusive subtyping initially, a core needle biopsy of the anterior mediastinal mass was performed. Histopathological examination confirmed TC features, and immunohistochemical profiling showed positivity for CD5 and CD117 (c-KIT), aligning with the bone marrow findings and confirming that the marrow involvement represented metastatic TC rather than a concurrent independent neoplasm.

Pathophysiologically, TC is a highly aggressive neoplasm driven by critical molecular alterations, such as TP53 mutations and inactivation of CDKN2A/CDKN2B, as well as epigenetic modifications that promote epithelial-mesenchymal transition, immune evasion, and neoangiogenesis [18]. These genomic aberrations confer a high-grade biological phenotype, characterized by a marked loss of thymic architecture, elevated mitotic activity, and an intrinsic propensity for early angiolymphatic invasion [19]. Unlike thymomas, TC exhibits a preference for systemic hematogenous dissemination [20]. In this context, we hypothesize that the pattern of diffuse bone marrow infiltration may be influenced by an anomalous tumor "homing" mechanism. Thymic neoplastic cells, after acquiring invasive mesenchymal characteristics, might interact with the bone marrow stromal microenvironment through chemotactic pathways dependent on the CXCR4/CXCL12 (SDF-1) axis [21,22]. This theoretical biological affinity could potentially contribute to liquid and diffuse marrow colonization, simulating the cellular kinetics of lymphoproliferative syndromes. Furthermore, extensive tissue replacement could explain or contribute to the onset of normocytic anemia, as well as the marked elevation of LDH and beta-2 microglobulin as indirect markers of tumor burden and cell turnover, rather than a proven mechanism in this specific patient.

Clinically, this case demonstrates that the absence of mediastinal symptoms does not exclude advanced thymic neoplasia, challenging the paradigm that these pathologies require initial thoracic manifestations. In the presence of refractory lumbar pain with analytical warning signs, it is imperative to perform early imaging extension studies. Timely recognition of this atypical metastatic phenotype is crucial in medical practice; it not only prevents erroneous diagnoses oriented toward hematological diseases but also allows for early referral to multidisciplinary management based on platinum-based regimens or immunotherapy to optimize the prognosis in advanced scenarios [23,24].

The main limitations of this report include the lack of molecular profiling (NGS) to confirm specific homing pathways, limited long-term follow-up, and the unavailability of the original computed tomography (CT) images from the chest and abdomen due to institutional archive constraints. Consequently, the proposed CXCR4/CXCL12 mechanism remains an explanatory hypothesis based on literature rather than a confirmed finding in this case. Nevertheless, diagnostic certainty was achieved through matched histopathological and immunohistochemical verification from both biopsy sites.

## Conclusions

Metastatic TC may present with diffuse bone marrow infiltration in the absence of respiratory symptoms, closely mimicking a lymphoproliferative neoplasm due to elevated beta-2 microglobulin and LDH levels. In patients presenting with refractory lumbar pain accompanied by red flags or unexplained laboratory abnormalities, considering additional imaging may be useful. Definitive diagnosis relies on histopathological confirmation with a targeted immunohistochemical panel; specifically, positivity for CD5, CD117, and pan-cytokeratin, combined with CD45 negativity, strongly supports the diagnosis in the context of mediastinal location and morphology, though it is not established by this marker combination alone. Recognizing this atypical presentation is crucial to avoiding diagnostic bias and facilitating appropriate diagnosis and management.
